# Association of serum 25-hydroxyvitamin D concentration and metabolic syndrome in Korean children and adolescents

**DOI:** 10.1186/1687-9856-2015-S1-P70

**Published:** 2015-04-28

**Authors:** Eun Young Kim, Tae Hwang, Kyung Hee Yi

**Affiliations:** 1 Department of Pediatrics, Chosun University Hospital, Gwangju, Korea; 2 Department of Pediatrics, Hallym University Kangdong Sacred Heart Hospital, Seoul, Korea; 3 Department of Pediatrics, Wonkwang University Sanbon Medical Center, Gunpo, Korea

## Aims

Vitamin D is required not only for bone health but also has been reported to play a role in a range of ailments such as autoimmune disease, cardiovascular disease, type 2 diabetes, hypertension, depression, and certain types of cancer. Several studies have reported that poor vitamin D status during childhood and adolescence is related to obesity and metabolic syndrome. Vitamin D deficiency has been associated with hypertension and increased risk of cardiovascular disease while 25(OH)D concentration is known to be independently associated with both insulin sensitivity and beta cell function. A recent study reported that low serum 25(OH)D to be associated with obesity and metabolic syndrome in Korean children. We investigated the association between serum 25(OH)D concentrations and the presence of metabolic syndrome components in Korean children and adolescents. We also compared the components of metabolic syndrome according to vitamin D status in Korean children and adolescents.

## Methods

The study included 141 Korean children and adolescents who were aged 6 to 18 in Kangdong Sacred Heart Hospital. Anthropometric measurements, including height, weight, body mass index (BMI, kg/m^2^) waist circumference, and blood pressure were performed. Serum lipid, fasting plasma glucose (FPG), insulin and 25(OH)D levels were measured. HOMA-IR (homeostasis model assessment for insulin resistance) = [glucose (mmol/L) x insulin]/22.5 values were calculated. Metabolic syndrome defined by modified National Cholesterol Education Program Adult Treatment Panel III (NCEP-ATP III).

## Results

Among total 141 subjects, 26 (18.4%) children and adolescents had metabolic syndrome. Children and adolescents who have metabolic syndrome have significantly lower serum 25(OH)D concentration than those who do not have (12.35 ± 4.65 vs. 14.81 ± 4.63 ng/mL) (p=0.015). Waist circumference, SBP, fasting plasma glucose, insulin, HOMA-IR are significantly correlated with serum 25(OH)D concentration (p<0.05). Only LDL-cholesterol is significantly different according to tertile groups of serum 25(OH)D concentration. The odds ratio of group I (25(OH)D <11.50 ng/mL) is 1.826 compared to group III (25(OH)D >16.30 ng/mL).

## Conclusion

Children and adolescents who have metabolic syndrome have lower serum 25(OH)D concentration. The prevalence of metabolic syndrome may be higher in children and adolescents with severe 25(OH)D deficiency. Further investigation will be needed to identify the influence of 25(OH)D on metabolic syndrome.

